# 2015 Update on Acute Adverse Reactions to Gadolinium based Contrast Agents in Cardiovascular MR. Large Multi-National and Multi-Ethnical Population Experience With 37788 Patients From the EuroCMR Registry

**DOI:** 10.1186/s12968-015-0168-3

**Published:** 2015-07-14

**Authors:** O. Bruder, S. Schneider, G. Pilz, A.C. van Rossum, J. Schwitter, D. Nothnagel, M. Lombardi, S. Buss, A. Wagner, S. Petersen, S. Greulich, C. Jensen, E. Nagel, U. Sechtem, H. Mahrholdt

**Affiliations:** Department of Cardiology and Angiology, Elisabeth Hospital, Essen, Germany; Institut für Herzinfarktforschung, Department of Biometrics, Ludwigshafen, Germany; Department of Cardiology, Hospital Agatharied, Hausham, Germany; Department of Cardiology, VU University Medical Centre, Amsterdam, The Netherlands; Centre Hospitalier Universitaire Vaudois - CHUV University of Lausanne, Lausanne, Switzerland; Department of Cardiology, Klinikum Ludwigsburg, Ludwigsburg, Germany; Fondazione C.N.R./Regione Toscana “G. Monasterio”, Pisa, Italy; Department of Cardiology, University of Heidelberg, Heidelberg, Germany; Cardiology Associates of Fairfield, Stamford, CT USA; Barts and The London NIHR Biomedical Research Unit, London Chest Hospital, London, United Kingdom; Division of Cardiovascular Imaging, J.W. Goethe University Frankfurt, Frankfurt am Main, Germany; German Center of Cardiovascular Research, Frankfurt, Germany; Department of Cardiology, Robert Bosch Medical Centre, Stuttgart, 70376 Germany

**Keywords:** Gadolinium, Safety, Cardiovascular magnetic resonance, “Off-label use”

## Abstract

**Objectives:**

Specifically we aim to demonstrate that the results of our earlier safety data hold true in this much larger multi-national and multi-ethnical population.

**Background:**

We sought to re-evaluate the frequency, manifestations, and severity of acute adverse reactions associated with administration of several gadolinium- based contrast agents during routine CMR on a European level.

**Methods:**

Multi-centre, multi-national, and multi-ethnical registry with consecutive enrolment of patients in 57 European centres.

**Results:**

During the current observation 37788 doses of Gadolinium based contrast agent were administered to 37788 patients. The mean dose was 24.7 ml (range 5–80 ml), which is equivalent to 0.123 mmol/kg (range 0.01 - 0.3 mmol/kg). Forty-five acute adverse reactions due to contrast administration occurred (0.12 %). Most reactions were classified as mild (43 of 45) according to the American College of Radiology definition. The most frequent complaints following contrast administration were rashes and hives (15 of 45), followed by nausea (10 of 45) and flushes (10 of 45). The event rate ranged from 0.05 % (linear non-ionic agent gadodiamide) to 0.42 % (linear ionic agent gadobenate dimeglumine). Interestingly, we also found different event rates between the three main indications for CMR ranging from 0.05 % (risk stratification in suspected CAD) to 0.22 % (viability in known CAD).

**Conclusions:**

The current data indicate that the results of the earlier safety data hold true in this much larger multi-national and multi-ethnical population. Thus, the “off-label” use of Gadolinium based contrast in cardiovascular MR should be regarded as safe concerning the frequency, manifestation and severity of acute events.

## Condensed abstract

We specifically aim to demonstrate that the results of our earlier safety data hold true in this much larger multi-national and multi-ethnical population. During the current observation 37788 doses of Gadolinium based contrast agent were administered to 37788 patients in a multi-centre, multi-national, and multi-ethnical setting with consecutive enrolment of patients in 57 European centres. Forty-five acute adverse reactions due to contrast administration occurred (0.12 %). Most reactions were classified as mild (43 of 45) according to the American College of Radiology definition. Thus, the “off-label” use of Gadolinium based contrast in cardiovascular MR should be regarded as safe concerning the frequency, manifestation and severity of acute events.

## Background

In its early phase the EuroCMR Registry concluded in 2011 on the basis of 17767 mostly German pilot patients [1] that the incidence of acute adverse reactions after administration of Gadolinium based contrast in the “off-label” setting of cardiovascular MR was not different to the incidence of acute adverse reactions in the FDA approved general radiology setting. Thus, on the basis of this data the “off-label” use of Gadolinium based contrast in cardiovascular MR was regarded as safe concerning the frequency, manifestation and severity of acute events.

In the meantime more than 37000 consecutive patients from 57 European centres in 15 countries have been included in the EuroCMR Registry. With the current update analysis we sought to re-evaluate the frequency, manifestations, and severity of acute adverse reactions associated with administration of several gadolinium- based contrast agents of routine CMR on a European level. Specifically, with this update we aim to demonstrate that the results of our earlier safety data [1] hold true in this much larger multi-national and multi-ethnical population.

## Methods

### Study population and data management

The current manuscript is based on data from the EuroCMR registry including 11040 consecutive patients of the German pilot phase (April 2007 and January 2009) and 31031 patients of the on going European registry (March 2009 - December 2014). All data were collected prospectively using online case record forms provided by the “Institut für Herzinfarktforschung Ludwigshafen”, University of Heidelberg, Germany (www.herzinfarktforschung.de), as previously described [1–4]. The local ethics committee approved all procedures with regard to data collection and management. A senior cardiologist or radiologist was appointed as local investigator and was responsible for the data quality of each patient entered in the registry in every centre.

### Analysis cohort

We included all patients receiving Gadolinium contrast in the analysis (n = 37788, 90 % out of 42071 patients). Gadolinium contrast was administered in compliance with the current ACCF/ACR/SCCT/SCMR/ASNC/NASCI/SCAI/SIR appropriateness criteria for CMR [2–5]. The medical records of all patients suspected to have suffered from an adverse event were reviewed centrally by a group of authors serving as an end-point committee.

### Variables and definitions

The EuroCMR Registry investigators collected pre-defined variables directly from patients, and/or from medical records, such as demographic data, history, and indications for CMR, procedural parameters, as well as complications [2–4]. As in our previous dataset [1], all complications caused by acute adverse reactions to contrast media (= onset within 60 minutes after administration) were defined according to the American College of Radiology [6] criteria (Table 1).

**Table 1:** Classification of severity and manifestation of adverse reactions to contrast media

**Mild**	
Signs and symptoms appear self-limited without evidence of progression (e.g., limited urticaria with mild pruritis, transient nausea, one episode of emesis) and include:	
• Nausea, vomiting	• Pallor
• Cough	• Flushing
• Warmth	• Chills
• Headache	• Sweats
• Dizziness	• Rash, hives
• Shaking	• Nasal stuffiness
• Altered taste	• Swelling: eyes, face
• Itching	• Anxiety
*Treatment:* Requires observation to confirm resolution and/or lack of progression and may require treatment in some cases. Patient reassurance is usually helpful.	
**Moderate**	
Signs and symptoms are more pronounced. Moderate degree of clinically evident focal or systemic signs or symptoms, including:	
• Tachycardia/bradycardia	• Bronchospasm, wheezing
• Hypertension	• Laryngeal edema
• Generalized or diffuse erythema	• Mild hypotension
• Dyspnea	
*Treatment:* Clinical findings in moderate reactions frequently require prompt treatment. These situations require close, careful observation for possible progression to a life-threatening event.	
**Severe**	
Sign and symptoms are often life-threatening, including:	
• Laryngeal edema (severe or rapidly progressing)	• Convulsions
• Profound hypotension	• Unresponsiveness
• Clinically manifest arrhythmias	• Cardiopulmonary arrest
*Treatment:* Requires *prompt* recognition and aggressive treatment; manifestations and treatment frequently require hospitalization.	

### Statistics

Senior statistician Steffen Schneider PhD, chair of the Biometrics Department of the Institut für Herzinfarktforschung, Ludwigshafen, Germany, performed the statistical analysis. We described the distribution of the different contrast media and the rate of adverse reactions in the patient population by absolute numbers and percentages. Furthermore, medians (with ranges) and means (with standard deviation) were calculated to describe the characteristics of contrast media, as well as to characterize the patients with adverse reactions. Fisher’s exact test was used to compare categorical parameters. Continuous variables were compared by Wilcoxon rank-sum test. However, the low rates of adverse events preclude most significance tests and the absence of pre-specified statistical hypothesis in the registry allows descriptive comparisons in most cases only. All analyses were performed using the SAS statistical package, version 9.3 (Cary, North Carolina).

## Results

### Acute adverse reactions by severity

The current dataset includes 37788 doses of Gadolinium based contrast agent from different vendors, which were administered to 37788 patients undergoing cardiovascular MR in the participating centres of the EuroCMR Registry. The mean dose was 24.7 ml (range 5–80 ml), which is equivalent to 0.123 mmol/kg (range 0.01 - 0.3 mmol/kg). We observed 45 acute adverse reactions due to contrast administration occurring during and immediately after the CMR procedure (0.12 %). The rate of female patients in the subgroup of the 45 patients suffering from adverse reactions was 38 %, which was not significantly different from the rate of females in the entire population (34 %). Wilcoxon rank sum test could not reveal any relations between acute reactions and the dose of Gadolinium administered (p = 0.09). Most adverse reactions were classified as mild (Table 1), no moderate reactions were recorded and only two events were regarded as severe reactions. There were no deaths due to contrast administration (and no deaths due to CMR imaging), and we did not observe an accumulation of events in a single centre or a cluster of centres.

### Acute adverse reactions by type of contrast media

Acute adverse reactions for the following contrast media were evaluated by the EuroCMR Registry; Gadopentetate (e.g. Magnevist), Gadoteracid (e.g. Dotarem), Gadobenat (e.g. Multihance), Gadobutrol (e.g. Gadovist), Gadoteridol (e.g. Prohance), Gadodiamide (e.g. Omniscan). All other contrast media were summarized as “others”. Eighteen out of the 57 centres exclusively used one single contrast agent for all patients (Gadopentetate: n = 3, Gadoteracid: n = 3, Gadobenat: n = 0, Gadobutrol: n = 8, Gadoteridol: n = 2, Gadodiamide: n = 2). All other centres used at least two or more different contrast agents in their clinical routine.

All events categorised by severity and the specific contrast media can be viewed in Table 2. Between the different agents the rate of adverse events ranged from 0.05 % to 0.42 %. However, our data did not reveal any relation between the event rates and the specific characteristics of the different contrast agents, including structure or chelate stability (p = 0.096), see Table 3.

**Table 2:** Adverse Reactions Categorized by Severity and Agent

Agent	N° of Examinations	N° of Adverse Reactions			
		Mild	Moderate	Severe	Total
Gadopentetat (e.g. Magnevist)	12810	18 (0.14)	0	2 (0.02)	20 (0.16)
Gadoteracid (e.g. Dotarem)	4235	5 (0.12)	0	0	5 (0.12)
Gadobenat (e.g. Multihance)	706	3 (0.42)	0	0	3 (0.42)
Gadobutrol (e.g. Gadovist)	9378	9 (0.1)	0	0	9 (0.10)
Gadoteridol (e.g. Prohance)	1045	2 (0.19)	0	0	2 (0.19)
Gadodiamide (e.g. Omniscan)	6116	3 (0.05)	0	0	3 (0.05)
Other	3498	3 (0.09)	0	0	3 (0.09)
Total	37788	43 (0.11)	0	2 (0.01)	45 (0.12)

Note - Values in parentheses are percentages

**Table 3:** Adverse Reactions Categorized by Agents Specific Characteristics

Agent	Molecular Structure	I Ionic / Non-Ionic	Log K_therm_	Reactions (%)
Gadopentetate (e.g. Magnevist)	Linear	Ionic	22.1	0.16
Gadoteracid (e.g. Dotarem)	Cyclic	Ionic	25.8	0.12
Gadobenat (e.g. Multihance)	Linear	Ionic	22.6	0.42
Gadobutrol (e.g. Gadovist)	Cyclic	Non-Ionic	21.8	0.10
Gadoteridol (e.g. Prohance)	Cyclic	Non-Ionic	23.8	0.19
Gadodiamide (e.g. Omniscan)	Linear	Non-Ionic	16.9	0.05
Others	N/A	N/A	N/A	N/A

Log K_therm_, chelate stability; N/A, non applicable

Table 4 displays the characteristics of all patients with acute adverse reactions by contrast agent. Complaints after Gadolinium administration include rashes and hives (15 of 45), followed by nausea (10 of 45) and flushes (10 of 45). In the group of the 45 patients suffering from adverse events, two suffered anaphylactic reactions that were graded as severe events due to the combination of bronchospasm and profound hypotension. Those two patients were admitted as inpatients, and were initially treated with adrenaline, steroids and antihistamines. Of the remaining 43 patients suffering mild events 30 were admitted for short-term observation, 16 of them were treated with steroids and/or antihistamines as a precaution. All patients improved during treatment and could be discharged later. No one except the two anaphylactic patients needed to stay as inpatients due to the adverse contrast reaction.

**Table 4:** Patient Characteristics, Gadolinium Dose, and Characteristics of Reaction

	All	Gadopentetat (Magnevist)	Gadoteracid (Dotarem)	Gadobenat (Multihance)	Gadotbutrol (Gadovist)	Gadoteridol (Prohance)	Gadodiamide (Omniscan)	Other*
All	45	20	5	3	9	2	3	3
Age (years, mean)	51.9	50.8	48.2	68.0	49.6	38.0	62.0	56.0
SD	16.5	18.1	19.0	4.6	16.1	14.1	4.0	13.0
Median	54.0	54.0	53.0	69.0	42.0	38.0	62.0	56.0
Range	16-77	16-74	25-69	63-72	31-77	28-48	58-66	43-69
Sex								
Male (%)	62.2	75.0	60.0	33.3	44.4	50	66.7	66.7
Contrast dose (ml, mean)	20.6	22.5	24.8	13.7	13.2	14.3	20.0	23.0
SD	8.3	7.5	8.8	3.2	4.3	17.0	8.0	11.5
Median	20.0	22.5	28.0	15.0	14.0	28.0	20.0	27.0
Range	7-40	7-36	10-32	10-16	10-21	16-40	12-28	10-32
Category of reaction								
Mild	43	18	5	3	9	2	3	3
Moderate	0	0	0	0	0	0	0	0
Severe	2	2	0	0	0	0	0	0
Symptoms								
Allergic shock	2	2	0	0	0	0	0	0
Cardiac arrest	0	0	0	0	0	0	0	0
Dyspnoea	1	1	0	0	0	0	0	0
Bronchospasm	1	1	0	0	0	0	0	0
Nausea	10	3	0	1	2	0	2	2
Vomiting	6	3	0	0	0	0	2	1
Warmth	3	2	0	1	0	0	0	0
Headache	2	1	0	0	0	0	0	1
Dizziness	5	3	0	0	0	0	2	0
Altered taste	1	0	0	0	0	0	0	1
Itching	9	2	1	1	4	1	0	0
Flushing	10	2	1	1	6	0	0	0
Chills	2	1	0	0	1	0	0	0
Sweats	3	2	0	0	1	0	0	0
Rash/Hives	15	4	4	2	2	2	1	0
Swelling of eye/face	1	1	0	0	0	0	0	0
Anxiety	6	6	0	0	0	0	0	0
Treatment								
Observation	30	14	3	2	5	1	2	3
Antihistamines	18	4	3	2	4	1	3	1
Steroids	14	3	1	2	3	2	3	0
Adrenaline	2	2	0	0	0	0	0	0

*Dotarem is included in Other

### Acute adverse events by CMR indication

Looking at all adverse reactions categorised by the initial CMR indication, analysis revealed different event rates for each of the three main indications [2–4]. The rate of adverse reactions ranged from 0.05 % for the group of mostly healthy individuals undergoing stress CMR for risk stratification in suspected coronary artery disease to 0.22 % for patients undergoing non-stress CMR for work-up of myocardial viability in the setting of known coronary artery disease and heart failure (p = 0.001), (Table 5). Consequently, more contrast related adverse events occurred in the non-stress CMR group (e.g. evaluation of myocardial viability) than in the stress CMR group (e.g. risk stratification in suspected CAD), (p = 0.004).

**Table 5:** Adverse Reactions Categorized by Indication

	All	RS CAD	MYO/CMP	Viability
Severe	2/37788 (0,01%)	2/18840 (0,01%)	0/14106 (0%)	0/4037 (0%)
Mild	43/37788 (0,11%)	7/18840 (0,04%)	22/14106 (0,16%)	9/4037 (0,22%)
All	45/37788 (0,12%)	9/18840 (0,05%)	22/14106 (0,16%)	9/4037 (0,22%)

RS CAD, risk stratification in suspected coronary artery disease; MYO/CMP, work-up of suspected myocarditis or cardiomyopathy; Viability, work-up of myocardial viability in known coronary artery disease. See references 1- 4 for details

## Discussion

Just 45 of all 37788 patients included suffered from Gadolinium related acute adverse reactions. Most reactions were classified as mild, only two patients suffered severe anaphylactic reactions, had to be admitted as inpatients, and were initially treated with adrenaline, steroids and antihistamines. All patients improved during treatment, and could be discharged later. Our current dataset confirms results of earlier safety data [1] in a much larger multi-national, and multi-ethnical population, indicating that the “off-label” use of Gadolinium based contrast in cardiovascular MR should be regarded as safe concerning the frequency, manifestation, and severity of acute events.

### Cardiovascular MR in comparison to general radiology use

The relatively low number of patients not receiving contrast media in comparison to general radiology use is most likely explained by the fact that for the three most important CMR indications: 1) evaluation of ischemia, 2) evaluation of cardiomyopathy and myocarditis, 3) evaluation of myocardial viability in CAD, the administration of contrast is mandatory. According to earlier EuroCMR Registry results more than 80 % of CMR scans are ordered for one of those three indications [2, 4].

Our data demonstrate a rate of acute adverse reactions of 0.12 %. This is even lower that in our previous dataset [1] (0.12 % vs. 0.17 %), and also in line with the observations of the FDA approved general radiology use of Gadolinium contrast, demonstrating a range of acute adverse reactions from 0.04 % up to 2.2 % [7–15].

Importantly, mostly mild, no moderate, and only two severe reactions occurred (Table 1), whereas other groups reported up to 17.2 % of moderate [12], and up to 6.3 % of severe reactions [12] in the general radiology setting.

### Differences between groups

Event rates between the groups receiving different contrast agents ranged from 0.05 % to 0.42 %. This finding also supports our previous report [1], and compares favourably with the results of a retrospective analysis of the FDA Adverse Event Reporting System [14]. One possible explanation for the different individual reaction rates was suggested by Prince et al. [14]. The authors suspected differences between the molecular structure and chelate stability in the different agents as possible cause. However, as displayed in Table 3 this does not seem to play a relevant role for acute adverse reactions in our population (p = 0.096). Nevertheless, parameters like structure and chelate stability may have an effect on long-term complications (e.g. nephrogenic systemic fibrosis). Unfortunately, we do not have long-term follow-up data due the limitations of the registry approach.

In addition, we found different reaction rates depending on the indication for CMR (see Table 5), as well as when comparing groups of patients undergoing stress CMR vs. non-stress CMR. As speculated earlier [1], this finding is most likely explained by the different burden of disease in the different patient groups. Whereas basically healthy people undergoing stress CMR for risk stratification of suspected coronary artery disease have the lowest rate of adverse events, the in comparison sick group of patients presenting with congestive heart failure for assessment of myocardial viability in the setting of known coronary artery disease has the highest rate of events. On the basis of this finding, one may even speculate that in this group some of the often unspecific symptoms such as nausea or anxiety (see Tables 1 and 4), which had been interpreted as Gadolinium related symptoms, may also be due to the underlying disease (e.g. heart failure). In fact, the reaction rate truly caused by Gadolinium itself could be even lower than that currently reported.

### Limitations

When interpreting registry data it is important to keep in mind that a prospective randomized trial may be the best tool to prove a certain principle, but only a registry can reveal if the results of controlled trials hold true in the multi-national multi-centre clinical routine [2]. Nevertheless, as in our previous manuscript [1], we can still only provide data on acute reactions, since systematic long-term follow-up (e.g. with regard to nephrogenic systemic fibrosis (NSF)) is not available due to the registry structure of the dataset. However, cases of NSF are not very likely in our population, since all participating centres only scanned patients after evaluation of renal function in compliance with our registry protocol [2–4] and the ACCF/ACR/SCCT/SCMR/ASNC/NASCI/SCAI/SIR appropriateness criteria for CMR imaging [5].

### Clinical implications

Our results demonstrate that our earlier safety data [1] hold true in this much larger multi-national and multi-ethnical population. Acute Gadolinium contrast related complications are rare, and the event rate favourably compares to that reported in the literature in a general radiology setting [7–15]. Thus, it seems safe to assume that the current “off-label use” of Gadolinium contrast for CMR is not associated with an additional risk for patients. In fact, datasets such as the EuroCMR Registry may help facilitating future FDA approval for Gadolinium in the setting of CMR.

## Conclusion

The current data indicate that the results of the earlier safety data hold true in this much larger multi-national and multi-ethnical population. Thus, the “off-label” use of Gadolinium based contrast in cardiovascular MR should be regarded as safe concerning the frequency, manifestation and severity of acute events.
